# Homocysteine and cognitive function in depression: a systematic review and meta-analysis

**DOI:** 10.3389/fpsyt.2026.1798998

**Published:** 2026-05-13

**Authors:** Xiaotong Liu, Qingsong Liu, Jili Xu, Jie Wu

**Affiliations:** 1 Chengdu University of Traditional Chinese Medicine, Chengdu, Sichuan, China; 2 Hospital of Chengdu University of Traditional Chinese Medicine, Chengdu, Sichuan, China

**Keywords:** cognition, cognitive impairment, depression, homocysteine, meta-analysis

## Abstract

**Objective:**

This study aimed to assess the association between homocysteine (Hcy) levels and cognitive function in individuals with depression using a systematic review and meta-analysis.

**Methods:**

We searched PubMed, Web of Science, EMBASE, the Cochrane Library, China National Knowledge Infrastructure (CNKI), Wanfang Data, and the Chinese Science and Technology Journal Database (VIP) for studies reporting Hcy levels and cognitive outcomes in patients with depression from inception to December 2025. Methodological quality was assessed using the Agency for Healthcare Research and Quality (AHRQ) checklist, evidence certainty was evaluated using the GRADE approach, and meta-analyzes were performed using random-effects models in Stata 17.0.

**Results:**

A total of 13 studies involving 1,269 patients with depression were included. The meta-analysis showed a significant inverse association between Hcy levels and cognitive scores (*r* = −0.41, 95% CI: −0.57 to −0.22, *p* < 0.001). High Hcy levels were associated with poorer cognitive performance (SMD = −0.37, 95% CI: −0.62 to −0.13, *p* = 0.003). Patients with cognitive impairment (CogI) also had higher Hcy levels than cognitively normal (CogN) individuals (SMD = 2.44, 95% CI: 0.86 to 4.01, *p* = 0.003).

**Conclusions:**

Hcy levels are associated with CogI in patients with depression, and subgroup analyzes and leave-one-out sensitivity analyzes indicate that the direction of the association is generally consistent. Further prospective studies are needed to clarify the clinical relevance of Hcy and whether it may contribute to the assessment of cognitive impairment in depression.

**Systematic review registration:**

https://www.crd.york.ac.uk/prospero/, identifier CRD420251206956.

## 1 Introduction

Depressive disorders are common and highly disabling mental disorders that remain highly prevalent worldwide and impose a substantial disease burden (1, 2). Moreover, depression places a considerable socioeconomic burden on society. For example, the incremental economic burden of major depressive disorder in the United States reached approximately $333.7 billion in 2019, largely due to productivity losses (3). Beyond core affective symptoms, depression is frequently associated with cognitive deficits, including impairments in attention, executive function, and memory (4, 5). Notably, such CogI substantially reduces quality of life (6) and may increase the risk of dementia (7, 8). Therefore, identifying factors associated with CogI may improve understanding of risk patterns and support future research on assessment strategies.

Hcy, a key intermediate in methionine metabolism, has received growing attention in research on depression and neurological disorders (9). Observational studies have frequently reported elevated Hcy levels in individuals with depression compared with healthy controls (10), and hyperhomocysteinemia has been associated with a higher risk of depression (11, 12). From a mechanistic perspective, dysregulation of one-carbon metabolism has been implicated in depression and may affect methylation and monoaminergic neurotransmission (13). Beyond depression, Hcy is also considered a risk factor for cognitive decline and dementia (14, 15). Community-based prospective cohort studies have further suggested that higher plasma Hcy predicts incident dementia and Alzheimer’s disease (16). Hcy may contribute to CogI through excitotoxic and neurotoxic effects, oxidative stress, blood–brain barrier (BBB) disruption, and cerebrovascular pathology (15, 17–19). Taken together, Hcy may represent a biologically relevant correlate of CogI in depression.

Nevertheless, the empirical evidence remains mixed. Observational studies have suggested an association between higher Hcy levels and depressive symptoms or depression status (20, 21). However, other population-based studies have reported null findings. The Zutphen Elderly Study did not support an association between Hcy and depressive symptoms, and a long-term longitudinal cohort did not find consistent associations between Hcy and depressive symptoms or incident depression in older adults (22, 23). In addition, a Mendelian randomization study did not support a causal effect of genetically predicted Hcy on the risk of major depressive disorder (24). Findings for cognition are also inconsistent. A large community-based cohort did not demonstrate a robust relationship between total Hcy and cognitive decline (25). Collectively, these inconsistent findings highlight uncertainty about whether, and to what extent, Hcy is clinically relevant to CogI in depression.

To date, quantitative evidence on the association between Hcy levels and cognitive function in individuals with depression remains limited. Against this background, we conducted a systematic review and meta-analysis of observational studies to quantify this association and explore potential sources of between-study heterogeneity. We aimed to provide a clearer picture of the relationship between Hcy and cognitive function in depression and to highlight priorities for future research.

## 2 Materials and methods

The protocol for this systematic review was registered in the International Prospective Register of Systematic Reviews (PROSPERO; registration number CRD420251206956). This study was conducted in accordance with the PRISMA 2020 guidelines (26).

### 2.1 Search strategy

We systematically searched PubMed, Web of Science, EMBASE, the Cochrane Library, China National Knowledge Infrastructure (CNKI), Wanfang Data, and the Chinese Science and Technology Journal Database (VIP) from inception to December 18, 2025 for studies on Hcy and CogI in depression. We used a combination of controlled vocabulary terms (e.g., MeSH and Emtree) and free-text terms. The search strategy comprised three concept blocks combined with AND: (1) depression (e.g., depression, depressive disorder, depressive symptoms, depressed, depressive); (2) homocysteine (e.g., homocysteine, Hcy, hyperhomocysteinemia); and (3) cognition (e.g., cognition, cognitive function, cognitive disorder, cognitive dysfunction, cognitive impairment). The detailed search strategy is provided in **Supplementary File 1**.

### 2.2 Inclusion and exclusion criteria

Eligibility criteria were as follows: (1) human studies that enrolled patients with depression, diagnosed using standardized criteria or validated instruments; (2) studies in which circulating Hcy was measured; (3) studies that reported cognitive outcomes as continuous cognitive scores or CogI status.

Exclusion criteria were: (1) animal studies; (2) non-original publications; (3) unpublished studies, studies without an available full text, or English-language manuscripts that were not publicly accessible; (4) conference abstracts, meeting proceedings, poster abstracts, and case reports; and (5) studies in which depression was secondary to major neurological events or severe medical conditions.

### 2.3 Article screening and data extraction

Two researchers independently screened titles/abstracts and subsequently full texts according to the predefined eligibility criteria. Disagreements were resolved through discussion. If consensus could not be reached, a third researcher adjudicated.

We used a standardized data extraction form to collect detailed information from each included study, including the author, year, country/region, sample size, age and sex distribution, cognitive and depression assessment instruments, and data required to compute effect sizes. Two reviewers independently extracted data, and a third reviewer cross-checked the extracted information for completeness and accuracy. When multiple cognitive outcomes were reported within the same study, we extracted only one effect size for each quantitative synthesis to avoid double-counting the same sample. We prioritized a global or overall cognitive measure when available. If no overall cognitive index was reported, we selected a single outcome according to a prespecified hierarchy based on the most representative cognitive measure described in the study. In addition, cognitive outcomes were harmonized so that higher values consistently indicated better cognitive function before calculating effect sizes. When studies reported both raw data and adjusted model-based estimates, effect sizes for quantitative synthesis were derived from raw data to maximize comparability across studies. When information needed for effect size calculation was missing, we first derived it from available summary statistics where possible or contacted corresponding authors to request additional data when necessary. If key data could not be obtained, the study was not included in the relevant quantitative synthesis but was retained in the narrative synthesis.

### 2.4 Risk of bias assessment and certainty of evidence

The methodological quality of the included studies was independently assessed by two reviewers using the 11-item checklist for cross-sectional studies developed by the Agency for Healthcare Research and Quality (AHRQ) (27). Discrepancies were resolved through discussion. If consensus could not be reached, a third reviewer adjudicated. For each item, ratings were recorded as “Yes,” “No,” or “Unclear.” A score of 1 was assigned for “Yes,” and 0 for “No,” or “Unclear,” yielding a total score ranging from 0 to 11. Studies scoring 0–3 were considered low quality, 4–7 moderate quality, and 8–11 high quality (28). The certainty of evidence for each outcome was assessed using the GRADE approach and was rated as high, moderate, low, or very low.

### 2.5 Statistical analysis

All analyzes were performed in Stata 17.0. Given the anticipated clinical and methodological heterogeneity, pooled effect sizes were estimated using a random-effects model (REML) and reported with 95% confidence intervals (CIs). Heterogeneity was assessed using Cochran’s Q test and quantified using I² and τ². Tests of pooled effects were two-sided, with *p* < 0.05 considered statistically significant. To provide complementary evidence and account for differences in the data available across studies, we categorized studies into two groups according to the type of effect size reported and synthesized them separately:

#### 2.5.1 Studies reporting correlation coefficients

Correlation coefficients (*r*) were used as the effect size in these studies. During data extraction, cognitive outcomes were harmonized so that higher values consistently indicated better cognitive function. Raw correlation coefficients reported in the original studies were directionally aligned when necessary and then transformed to Fisher’s Z values and pooled using a random-effects model (29). After pooling on the Z scale, the summary estimate and its 95% CI were back-transformed to r for reporting and forest-plot presentation. The value of *r* ranges from −1 to 1; the sign indicates direction, and values closer to 0 indicate weaker associations. Correlations were interpreted as follows: 0–0.19 (no correlation), 0.20–0.39 (low correlation), 0.40–0.59 (moderate correlation), 0.60–0.79 (moderately high correlation), and ≥0.80 (high correlation) (30).

#### 2.5.2 Studies pooled as standardized mean differences

For group-comparison syntheses, SMDs were calculated from reported group summary statistics when available. This category included two types of between-group comparisons: (i) cognitive scores in high-Hcy versus low-Hcy groups and (ii) Hcy levels in participants with CogI versus CogN. For (i), SMDs were used to pool between-group differences in cognitive scores; after harmonizing effect directions, an SMD < 0 indicated poorer cognitive performance in the high-Hcy group. For (ii), SMDs were calculated as (Mean_impairment − Mean_normal)/SD_pooled. An SMD > 0 indicated higher Hcy levels in the CogI group.

Subgroup analyzes were conducted only when study-level information was sufficiently comparable and the number of studies was adequate to support meaningful stratification. Meta-regression was not undertaken in quantitative syntheses with a limited number of studies or insufficiently consistent reporting of study characteristics across the original reports.

## 3 Results

### 3.1 Study identification and selection

**Figure 1:**
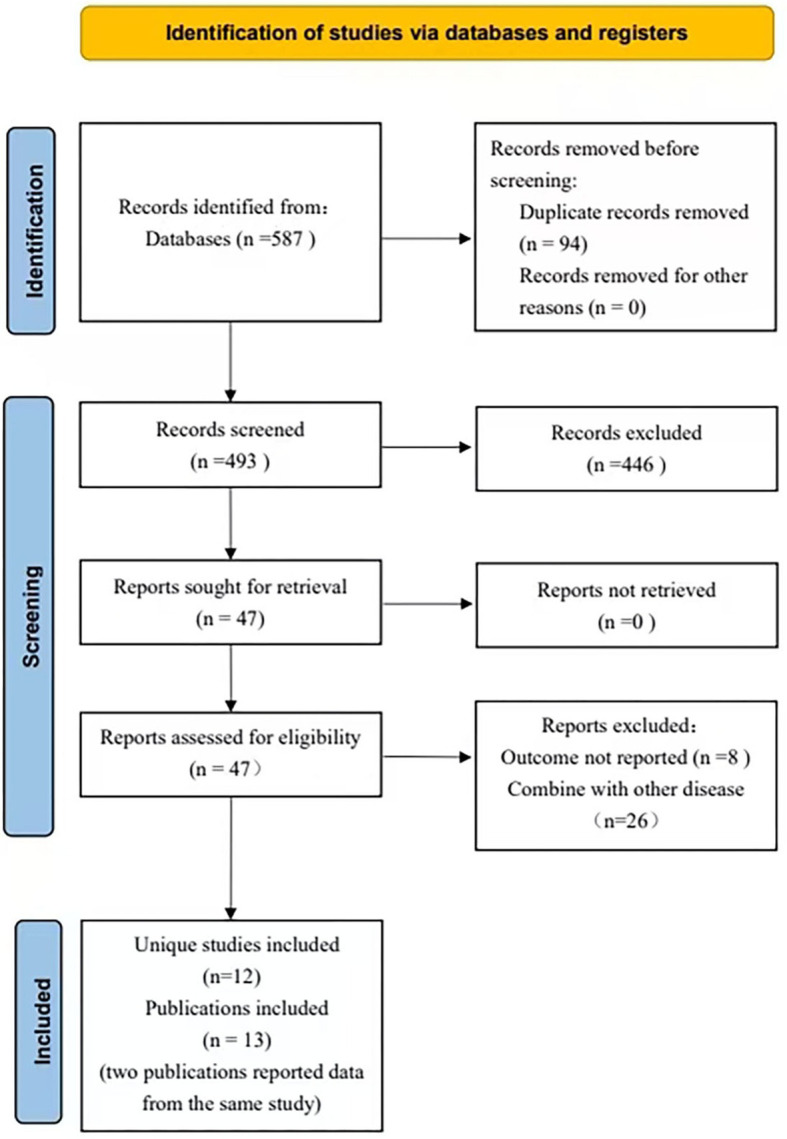
PRISMA flow chart. Flow chart based on PRISMA 2020 guidelines detailing the screening strategy employed for the study selection.

A total of 587 records were identified through the literature search. After removing duplicates, 493 records remained for title and abstract screening, and 446 were excluded. The full texts of 47 articles were retrieved and assessed for eligibility; 34 were excluded after full-text review. Ultimately, 13 articles were included (31–43). Two articles, Zhou et al. (2020) (38) and Zhou et al. (2021) (36), were derived from the same underlying study population. To avoid double counting, these two articles were treated as one independent study source in the quantitative synthesis, and only one effect size from this shared sample was included in each meta-analysis. When both articles were relevant to the same meta-analysis, we selected the one that could directly provide the effect estimate required for that specific synthesis. For example, in the correlation-based synthesis, we used Zhou et al. (2021) (36) because it reported data that could be directly incorporated into the pooled correlation analysis. Therefore, although 13 articles were included in the review, the quantitative synthesis was based on 12 independent studies (**Figure 1**).

### 3.2 Study characteristics

The main characteristics of the 12 included studies are summarized in **Table 1**. In total, 1,269 participants were included. Overall, women accounted for 51.94% of the sample and men for 48.06%. The mean age across studies ranged from 34.97 to 73.00 years; the sample size–weighted overall mean age was approximately 60.80 years. The studies were conducted in four countries, with the majority from China (9 studies) (31–37, 39, 43), and the remainder from Australia (40), Germany (41), and the United States (42) (1 study each). All included studies used a cross-sectional design, indicating that the quantitative synthesis was based only on cross-sectional evidence. Depression was primarily diagnosed according to the Diagnostic and Statistical Manual of Mental Disorders (DSM) or the International Classification of Diseases, 10th Revision (ICD-10) criteria, with some studies additionally applying cut-off scores on the Hamilton Depression Rating Scale (HAMD) or clinical diagnostic criteria. Cognitive function was most commonly assessed using the Mini-Mental State Examination (MMSE) (6 studies) (32–34, 36, 42, 43), followed by the Montreal Cognitive Assessment (MoCA) (3 studies) (35, 37, 39). Other instruments included the MATRICS Consensus Cognitive Battery (MCCB) (1 study) (31), the Consortium to Establish a Registry for Alzheimer’s Disease (CERAD) neuropsychological battery (1 study) (40), and the Stroop Color and Word Test combined with the d2 Test of Attention (1 study) (41).

**Table 1:** Characteristics of the included studies.

Study	Country	Study design	Sample size	Depression diagnostic criteria	Cognitive test	Mean age (SD)	M/F	Depression severity scale	Medication status	Main adjusted covariates
Xu, 2025	China	Cross-sectional study	67	ICD-10	MCCB	34.97	30/37	HAMD-17	Medication-free	Age, Sex
Fan, 2025	China	Cross-sectional study	60	DSM-5	MMSE	69.94	29/41	HAMD-17	Medication-free	Age, Sex, Education
Zhang, 2024	China	Cross-sectional study	177	Clinical	MMSE	50.44	119/58	HAMD	Medication-free	Not reported
Wang, 2024	China	Cross-sectional study	56	DSM-IV	MMSE	70.4	16/40	Not reported	Not reported	Not reported
Zhou, 2021	China	Cross-sectional study	113	DSM-IV	MMSE	67.6	26/87	HAMD	Not reported	Age, Sex, Education, BMI, Smoking
Gou2021	China	Cross-sectional study	100	HAMD-17	MoCA	36.9	58/42	HAMD-17	Not reported	Not reported
Zhu2020	China	Cross-sectional study	60	ICD-10	MoCA	65.82	26/34	HAMD	Not reported	Not reported
/lHuang, 2020	China	Cross-sectional study	116	ICD-10	MMSE	69.12	67/49	HAMD	Medication-free	Not reported
Guo, 2024	China	Cross-sectional study	206	ICD-10	MoCA	68.68	115/91	HAMD-17	Not reported	Not reported
Andrew, 2013	Australia	Cross-sectional study	236	DSM-IV	CERAD	62.34	107/129	Not reported	Unclear	Age, Sex, IHD
Alexopoulos, 2010	Germany	Cross-sectional study	65	DSM-IV	Stroop Test、d2 Test	71.48	16/49	HAMD、TE4D	On medication	Age, Sex, Education
Bell, 1992	America	Cross-sectional study	13	DSM-III-R	MMSE	73	1/12	Not reported	Not reported	Not reported

For studies reporting age by subgroups only, overall mean age (and SD, when applicable) was calculated using sample size - weighted pooling; M/F, Male/Female; IHD, Ischemic heart disease.

### 3.3 Quality assessment

The methodological quality assessment of the included studies is presented in **Table 2**, which summarizes the item-level judgments for each study across the assessed quality domains and the corresponding overall quality classifications. Overall, among the 12 included studies, 7 were rated as high quality and 5 as moderate quality. The certainty of evidence was assessed using the GRADE approach, and was generally rated as low (**Supplementary File 2**).

**Table 2:** Methodological quality for included study.

Study	1)	2)	3)	4)	5)	6)	7)	8)	9)	10)	11)	Score	Overall quality
Xu, 2025	Yes	Yes	Yes	No	No	Yes	No	Yes	Yes	Yes	Yes	8	High
Fan, 2025	Yes	Yes	No	No	No	Yes	No	Yes	Yes	Yes	Yes	7	Moderate
Zhang, 2024	Yes	Yes	Yes	No	No	Yes	No	Yes	No	Yes	Yes	7	Moderate
Wang, 2024	Yes	Yes	Yes	No	Yes	Yes	Yes	Yes	No	Yes	Yes	9	High
Gou, 2021	Yes	Yes	Yes	No	No	No	No	Yes	No	Yes	Yes	6	Moderate
Zhou, 2021	Yes	Yes	Yes	No	No	No	No	Yes	No	Yes	Yes	6	Moderate
Zhu, 2020	Yes	Yes	Yes	No	No	Yes	No	No	Yes	Yes	Yes	7	Moderate
Zhou, 2020	Yes	Yes	No	Yes	No	Yes	No	Yes	Yes	Yes	Yes	8	High
Guo, 2014	Yes	Yes	Yes	No	No	Yes	No	Yes	Yes	Yes	Yes	9	High
Andrew, 2013	Yes	Yes	Yes	Yes	No	Yes	No	Yes	Yes	Yes	Yes	9	High
Alexopoulos, 2010	Yes	Yes	Yes	Yes	No	Yes	No	Yes	Yes	Yes	Yes	9	High
Bell, 1992	Yes	Yes	Yes	Yes	No	Yes	No	Yes	Yes	Yes	Yes	9	High

Although 13 articles were included in this review, only 11 reported data that could be converted into a common effect size and were therefore eligible for quantitative synthesis. The remaining two articles could not be pooled because their outcome reporting was not comparable with that of the other studies; these were summarized narratively (38, 39).

### 3.4 Meta-analyzes

#### 3.4.1 Correlation between Hcy and cognitive function

**Figure 2:**
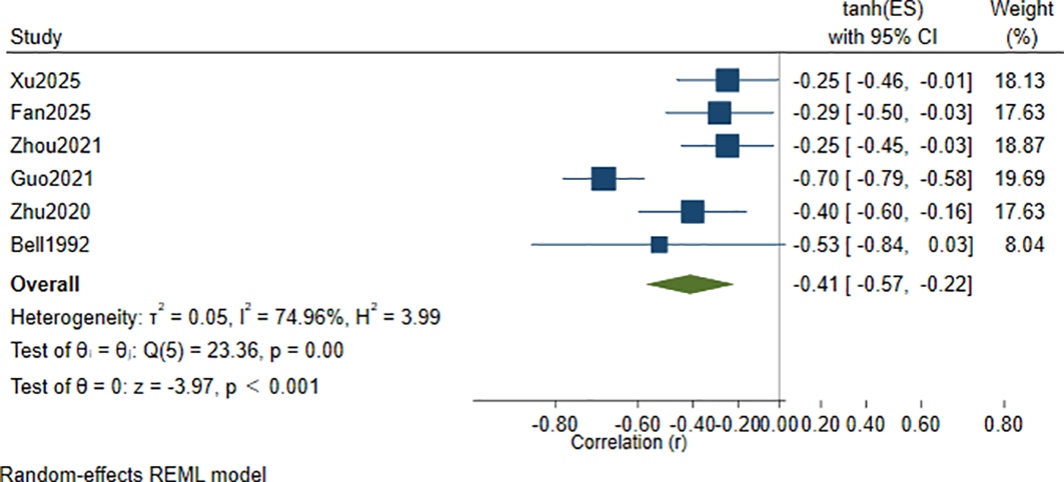
Forest plot of the correlation between serum Hcy levels and cognitive function scores in patients with depression.

A meta-analysis of the correlation coefficients reported in the included studies (31, 32, 35–37, 42) showed a significant inverse association between serum Hcy levels and cognitive function scores, with a pooled correlation of *r* = −0.41 (95% CI: −0.57 to −0.22, *p* < 0.001), with *I*² = 74.96% and *τ*² = 0.05 (**Figure 2**). To explore potential sources of between-study variability, we further conducted subgroup analyzes.

**Figure 3:**
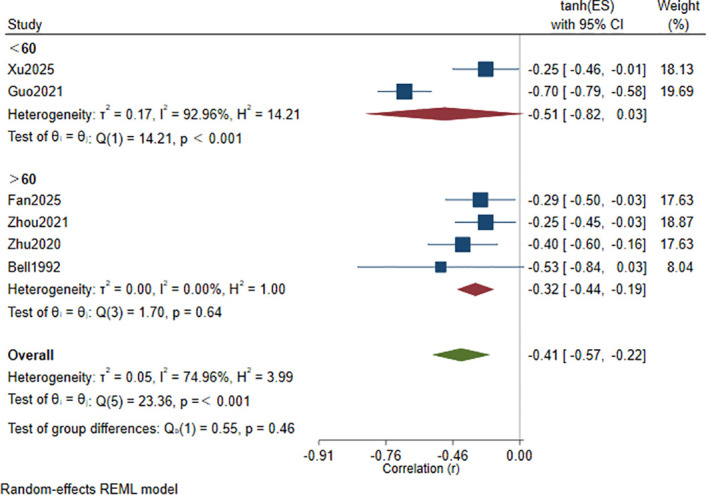
Subgroup analysis by age of the correlation between Hcy levels and cognitive function scores.

Age-stratified analysis (**Figure 3**):

In the subgroup of participants aged <60 years (31, 35), the pooled correlation was *r* = −0.51 (95% CI: −0.82 to 0.03), with *I*² = 92.96% and *τ*² = 0.17. In the subgroup aged ≥60 years (32, 36, 37, 42), the pooled correlation was *r* = −0.32 (95% CI: −0.44 to −0.19), with *I*² = 0.00% and *τ*² = 0.00. The direction of the association was consistent across subgroups, and both showed an inverse relationship. However, the between-subgroup difference was not statistically significant (p = 0.46).

**Figure 4:**
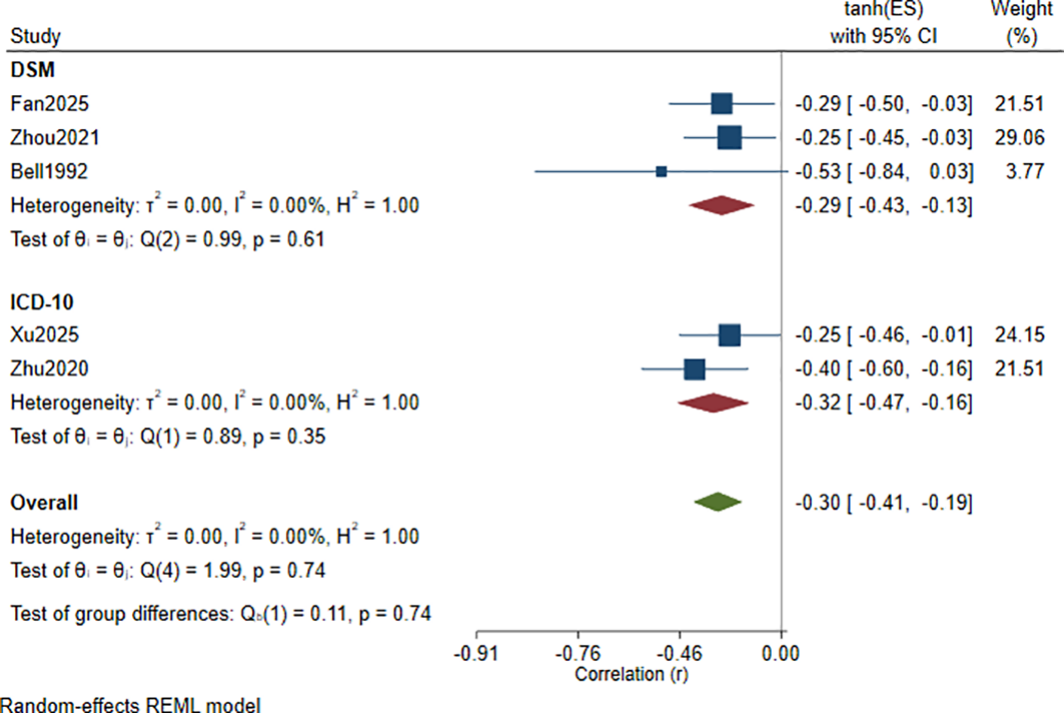
Subgroup analysis by diagnostic criteria for depression of the correlation between Hcy levels and cognitive function scores.

Stratified by diagnostic criteria for depression (**Figure 4**):

The pooled correlation in the DSM subgroup (32, 36, 42) was *r* = −0.29 (95% CI: −0.43 to −0.13), and the pooled correlation in the ICD-10 subgroup (31, 37) was *r* = −0.32 (95% CI: −0.47 to −0.16). The direction and magnitude of the associations were similar across subgroups, and the between-subgroup difference was not statistically significant (*p* = 0.74). Heterogeneity was the same in the two subgroups, with *I*² = 0.00% and *τ*² = 0.00.

**Figure 5:**
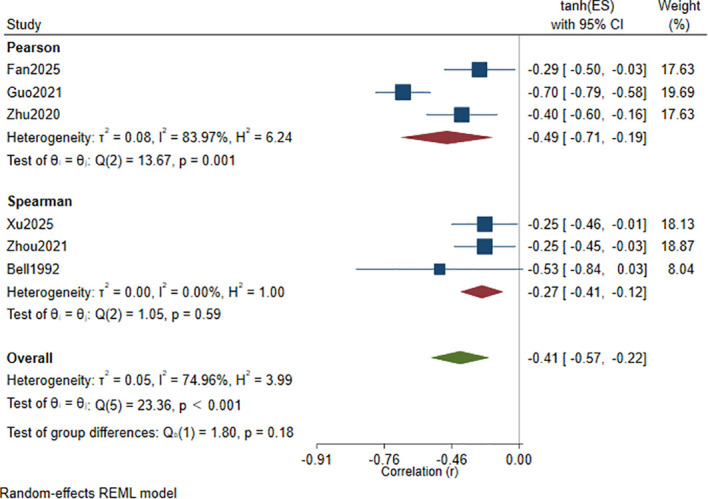
Subgroup analysis by correlation type of the correlation between Hcy levels and cognitive function scores.

Stratified by correlation type (**Figure 5**):

The pooled correlation in the Pearson subgroup (32, 35, 37) was *r* = −0.49 (95% CI: −0.71 to −0.19), with *I*² = 83.97% and *τ*² = 0.08, whereas the pooled correlation in the Spearman subgroup (31, 36, 42) was *r* = −0.27 (95% CI: −0.41 to −0.12), with *I*² = 0.00% and *τ*² = 0.00. Both subgroups showed an inverse association, and the between-subgroup difference was not statistically significant (*p* = 0.18).

**Figure 6:**
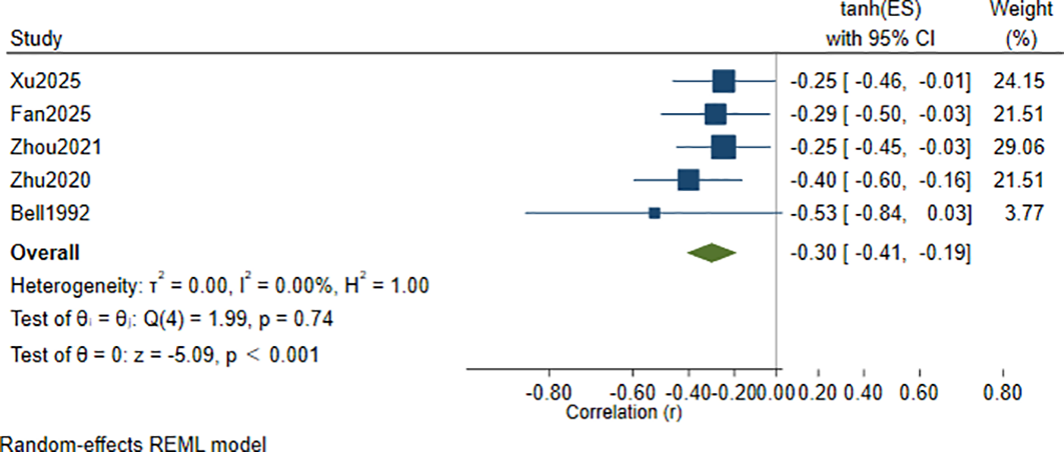
Leave-one-out sensitivity analysis excluding Guo et al. (2021): forest plot of the correlation between Hcy levels and cognitive function scores.

Leave-one-out sensitivity analysis identified Guo et al. (2021) (35) as an influential study. After excluding this study, the pooled correlation decreased to *r* = −0.30 (95% CI: −0.41 to −0.19), and heterogeneity was substantially reduced, with *I*² = 0.00% and *τ*² = 0.00 (**Figure 6**; **Supplementary File 3**). Due to the small number of included studies (<10), funnel plot analysis was not performed, as it would be insufficiently powered to detect publication bias. Egger’s test and trim-and-fill analysis were also not undertaken for the same reason.

#### 3.4.2 Standardized mean difference in cognitive scores between high-Hcy and low-Hcy groups

**Figure 7:**
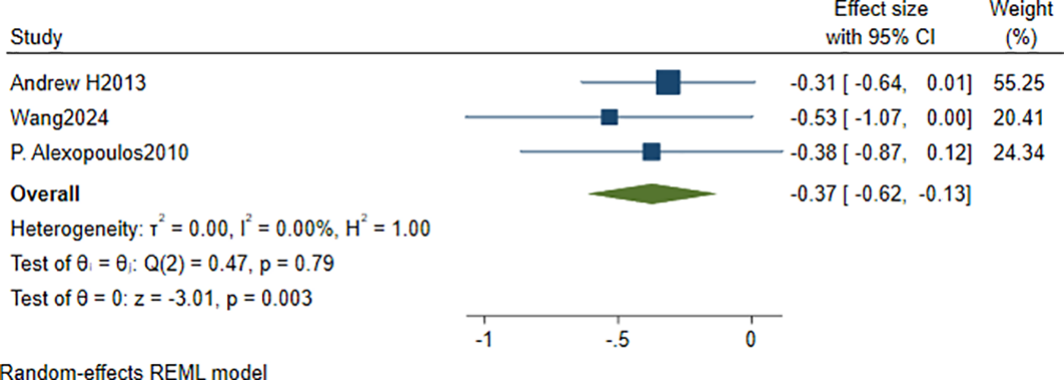
Forest plot of differences in cognitive scores between high-Hcy and low-Hcy groups in patients with depression.

Three studies (34, 40, 41) were included to compare cognitive scores between high-Hcy and low-Hcy groups in patients with depression. The pooled analysis showed that cognitive scores were significantly lower in the high-Hcy group than in the low-Hcy group (SMD = −0.37, 95% CI: −0.62 to −0.13, *p* = 0.003), with *I*² = 0.00% and *τ*² = 0.00 (**Figure 7**).

#### 3.4.3 Standardized mean difference in Hcy levels between CogI and CogN

**Figure 8:**
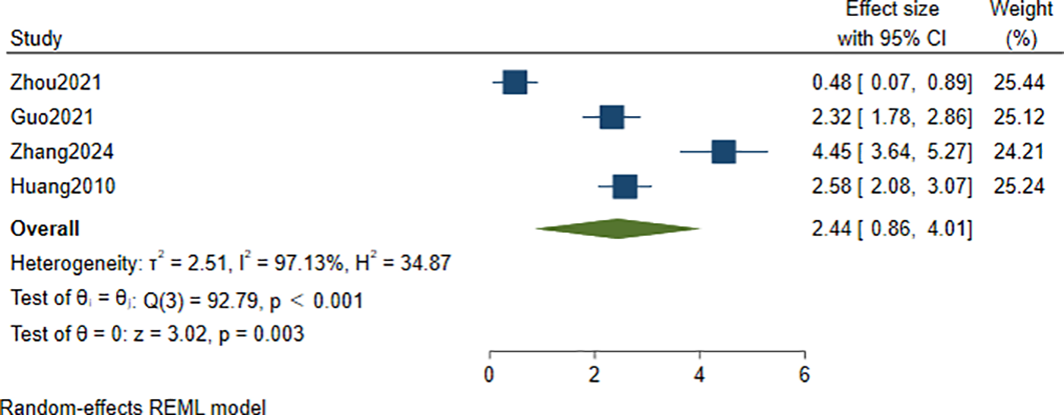
Forest plot of differences in Hcy levels between patients with CogI and those with Cog.

Four studies (33, 35, 36, 43) compared Hcy levels between patients with CogI and those with CogN. The pooled results indicated significantly higher Hcy levels in the CogI group (SMD = 2.44, 95% CI: 0.86 to 4.01, *p* = 0.003), with *I*² = 97.13% and *τ*² = 2.51 (**Figure 8**).

### 3.5 Narrative synthesis of studies not included in meta-analysis

Two studies were not included in the quantitative synthesis because their effect measures were not compatible with the prespecified meta-analytic approach. These studies were therefore summarized narratively as supplementary evidence.

Zhou et al. (2020) (38) reported an inverse association between homocysteine levels and cognitive function in older adults with depression. The authors also reported a significant interaction between elevated Hcy and depressive status, suggesting more pronounced CogI when both factors were present. Because this study primarily reported regression coefficients and interaction terms without providing effect estimates that could be harmonized for pooling, it was not included in the meta-analysis.

Another cross-sectional study in older adults with depression (39) used logistic regression to examine factors associated with CogI and found that elevated Hcy was independently associated with CogI. Because this study primarily reported categorical outcomes and odds ratios as effect measures, it was likewise not included in the quantitative synthesis.

Overall, the findings of these studies were consistent in direction with our meta-analytic results and further supported an association between elevated Hcy levels and CogI in patients with depression.

## 4 Discussion

This systematic review and meta-analysis synthesized the available evidence, indicating a consistent association between Hcy levels and cognitive performance in patients with depression. The pooled correlation analysis showed that higher Hcy levels were associated with poorer cognitive scores (*r* = −0.41, 95% CI: −0.57 to −0.22). Consistent with these findings, meta-analyzes based on between-group comparisons supported the same pattern. Cognitive scores were lower in high-Hcy groups than in low-Hcy groups (SMD = −0.37, 95% CI: −0.62 to −0.13), and Hcy levels were higher in individuals with cognitive impairment (CogI) than in cognitively normal individuals (CogN) (SMD = 2.44, 95% CI: 0.86 to 4.01). Taken together, these findings across different effect-size metrics suggest that Hcy may be associated with CogI in patients with depression.

Leave-one-out sensitivity analyzes showed that the direction of the pooled association remained generally unchanged when any single study was removed, which supported the robustness of the main findings. However, excluding Guo et al. (2021) led to a marked reduction in between-study heterogeneity and a smaller pooled effect size, suggesting that this study was a major contributor to overall heterogeneity. This influence may relate to differences in sample characteristics and within-study phenotypic stratification. Specifically, participants in Guo et al. (2021) were younger on average. Their baseline vascular and metabolic burden, comorbidity profiles, and depression phenotypes may also have differed from those of the mainly middle-aged and older participants in the other studies, which may have influenced the strength of the Hcy–cognition association (44–46). In addition, Guo et al. (2021) stratified the analyzes by depression severity and observed a clearer gradient in both Hcy levels and cognitive scores across severity strata. This pattern may have strengthened the observed association and made the study more influential in the pooled estimate.

Although the present meta-analysis does not permit direct mechanistic inference, several biological pathways have been proposed in previous experimental and clinical studies that may help explain the observed association between Hcy and cognitive outcomes in depression. First, Hcy is a key component of one-carbon metabolism and the methylation network. Elevated Hcy has been linked to reduced S-adenosylmethionine (SAM), which may result in insufficient methyl-donor availability and subsequently disrupt the metabolism and function of monoaminergic neurotransmitters (13), such as dopamine, 5-hydroxytryptamine, and norepinephrine. This may contribute to both affective and cognitive symptoms. Second, Hcy may affect synaptic transmission and neuroplasticity through NMDA receptor–related excitatory pathways and neurotoxic effects, potentially reducing cognitive efficiency (47, 48). In addition, Hcy oxidation can increase reactive oxygen species production and oxidative stress and has also been associated with increased inflammatory activity. Inflammation and oxidative stress may further impair neuronal integrity and cognitive function (49, 50). Because the included studies mainly involved middle-aged and older adults, vascular mechanisms may be particularly relevant in this population, including endothelial dysfunction, cerebral small-vessel disease, and BBB disruption (51, 52). Previous evidence also links elevated Hcy to BBB dysfunction, neuroinflammation, and white matter abnormalities (53, 54), which may provide a biological basis for CogI comorbid with depression. Finally, animal studies and some clinical data suggest an association between higher Hcy levels and lower neurotrophic factors, such as brain-derived neurotrophic factor (BDNF). This may contribute to cognitive impairment in depression by reducing neuroplasticity. However, more studies in depressed populations are needed to clarify causality, direction, and clinical relevance (55–57).

Regarding potential modifiers, we conducted subgroup analyzes within the correlation meta-analysis by age, diagnostic criteria for depression, and correlation type, and did not observe statistically significant between-subgroup differences. However, this lack of statistically significant subgroup differences should not be interpreted as evidence that these factors do not modify the association between Hcy and cognitive outcomes in depression. Rather, it is more likely to reflect the limited number of available studies, insufficient statistical power, and substantial methodological heterogeneity across studies. Previous research has suggested that the magnitude of the association between Hcy and depression-related cognitive outcomes may vary across populations and study settings. For example, the coexistence of elevated Hcy and poorer cognitive performance has been reported to be more pronounced in late-life depression (38). In addition, a prior systematic review indicated that differences in the instruments used to assess or diagnose depression may influence the observed association between Hcy and depression-related outcomes (21). Methodological variation in the present literature, including differences in cognitive assessment tools, laboratory methods for Hcy measurement, and the degree of adjustment for confounding, may also have obscured potential effect modification (58, 59). Furthermore, other clinically relevant sources of heterogeneity were likely present across the included studies, such as differences in depression subtype, treatment exposure, and comorbidity burden. Because these characteristics were reported inconsistently and the number of studies was limited, they could not be examined in robust subgroup analyzes or meta-regression. Accordingly, the current evidence is insufficient to draw firm conclusions about moderators of the association between Hcy and cognitive outcomes in depression. Future studies should include larger and better-characterized samples, adopt more standardized approaches to cognitive and biomarker assessment, and use prespecified stratified analyzes to determine whether age, clinical phenotype, treatment status, and comorbidity profiles systematically influence the strength of this association.

Given that most included studies were cross-sectional, the observed association between Hcy and cognitive outcomes should be interpreted as an association rather than evidence of causality. At present, Hcy should not be regarded as a diagnostic marker for cognitive impairment in depression or as a stand-alone basis for clinical decision-making. Because the present study did not assess diagnostic performance, threshold values, sensitivity, specificity, or predictive accuracy, the current evidence is insufficient to establish the clinical utility of Hcy in this context. Nevertheless, the present meta-analysis remains clinically and scientifically informative by showing a consistent association between elevated Hcy and poorer cognitive outcomes in depression. These findings suggest that Hcy warrants further investigation as a potentially relevant correlate of cognitive impairment in depression (60). In light of the current evidence, future research should focus on several priorities. Large prospective studies with comprehensive adjustment for key confounders are needed to determine whether the association between Hcy and cognitive outcomes in depression is independent. In addition, future work should examine whether Hcy provides incremental information when evaluated together with other relevant biomarkers in multivariable models (61–63). Findings from randomized trials in broader populations on whether lowering Hcy improves cognition have been inconsistent (64, 65). Therefore, randomized controlled trials in depressed populations will also be important to determine whether modifying Hcy influences cognitive outcomes and whether any such effect is clinically meaningful.

Several limitations warrant consideration. Because the included studies were predominantly cross-sectional, both causal inference and interpretation of the pooled findings require caution. Considerable heterogeneity in study populations and measurement methods may affect the comparability of the pooled estimates. Some results appeared sensitive to individual studies, and the evidence base was skewed toward middle-aged and older populations, which may limit generalizability to younger individuals with depression. In addition, the small number of studies restricted subgroup analyzes and precluded formal assessment of publication bias. Importantly, Hcy levels are influenced by multiple factors beyond depression status itself, including age, renal function, folate and vitamin B12 status, cardiovascular risk burden, and lifestyle factors such as smoking and diet. Because these variables were not consistently reported or adjusted for across the included studies, residual confounding cannot be excluded when interpreting the observed association between Hcy and cognitive outcomes. In addition, because the quantitative synthesis was based mainly on raw and unadjusted estimates, the pooled associations should not be interpreted as evidence of an independent effect of Hcy.

## 5 Conclusion

This systematic review and meta-analysis found that elevated Hcy levels were associated with poorer cognitive performance in patients with depression. Consistent findings across correlation and between-group analyzes indicated a higher likelihood of CogI among individuals with increased Hcy levels. However, given the predominantly cross-sectional design, the largely observational and mainly crude nature of the available evidence, and heterogeneity across studies, causal inference and clinical utility remain uncertain. Further prospective studies are warranted to clarify the potential clinical relevance of Hcy in depression-related CogI and whether it may contribute useful information to cognitive assessment in this population.

## Data Availability

The original contributions presented in the study are included in the article/**Supplementary Material**. Further inquiries can be directed to the corresponding author.
