# Supplementary material for: Homocysteine and cognitive function in depression: a systematic review and meta-analysis
Source: Front Psychiatry. 2026 May 13;17:1798998. doi: 10.3389/fpsyt.2026.1798998 (PMC13212284; doi:10.3389/fpsyt.2026.1798998)
Supplement: Supplementary file 1 [file Table1.docx]

**Supplementary File 1:** Search strategy

| Pubmed： |
| --- |
| 1. "Depression"[Mesh] 2. ((depression[Title/Abstract]) OR ("depressive disorder"[Title/Abstract]) OR ("depressive symptoms"[Title/Abstract]) OR (depressed[Title/Abstract]) OR (depressive[Title/Abstract])) 3. #1 or #2 4. "Homocysteine"[Mesh] 5. ((homocysteine[Title/Abstract]) OR (Hcy[Title/Abstract]) OR (hyperhomocysteinemia[Title/Abstract])) 6. #4 or #5 7. "Cognition"[Mesh] 8. ((cognition[Title/Abstract]) OR ("cognitive function"[Title/Abstract]) OR ("cognitive disorder"[Title/Abstract]) OR ("cognitive dysfunction"[Title/Abstract] OR ("cognitive impairment"[Title/Abstract] )) 9. #7 or #8 10. #3 AND #6 AND #9 |
| Web of Science: |
| 1. TS=(depression OR depressive disorder OR depressive symptoms OR depressed OR depressive) 2. TS=(homocysteine OR Hcy OR tHcy OR hyperhomocysteinemia) 3. TS=(cognition OR cognitive function OR cognitive disorder OR cognitive dysfunction OR cognitive impairment ) 4. #1 AND #2 AND #3 |
| EMBASE： |
| 1. 'depression'/exp 2. 'depressive disorder'/exp 3. depression:ab,ti OR 'depressive disorder':ab,ti OR 'depressive symptoms':ab,ti OR depressed:ab,ti OR depressive:ab,ti 4. #1 OR #2 OR #3 5. 'homocysteine'/exp 6. homocysteine:ab,ti OR hcy:ab,ti OR hyperhomocysteinemia:ab,ti 7. #5 OR #6 8. 'cognition'/exp 9. 'cognitive dysfunction'/exp 10. cognition:ab,ti OR 'cognitive function':ab,ti OR 'cognitive disorder':ab,ti OR 'cognitive dysfunction':ab,ti OR 'cognitive impairment':ab,ti 11. #8 OR #9 OR #10 12. #4 AND #7 AND #11 |
| Cochrane Library |
| 1.MeSH descriptor: [Depression] explode all trees  2.MeSH descriptor: [Depressive Disorder] explode all trees  3.(depression OR depressive disorder OR depressive symptoms OR depressed OR depressive):ti,ab,kw  4.#1 OR #2 OR #3  5. MeSH descriptor: [Homocysteine] explode all trees  6. (homocysteine OR Hcy OR hyperhomocysteinemia):ti,ab,kw  7. #5 OR #6  8. MeSH descriptor: [Cognition] explode all trees  9. MeSH descriptor: [Cognitive Dysfunction] explode all trees  10. (cognition OR cognitive function OR cognitive disorder OR cognitive dysfunction):ti,ab,kw  11. #8 OR #9 OR #10  12. #4 AND #7 AND #11 |
| CNKI |
| SU=('抑郁' + '抑郁症' + '抑郁状态' + '抑郁情绪') AND SU=('同型半胱氨酸' + '同型半胱氨酸血症' + 'Hcy') AND SU=('认知' + '认知功能' + '认知损害') |
| Wanfang Data |
| 主题:(抑郁 or 抑郁症 or 抑郁状态 or抑郁情绪) and 主题:(同型半胱氨酸 or 同型半胱氨酸血症 or Hcy) and 主题:(认知 or 认知功能 or 认知障碍 or 认知损害) |
| VIP |
| (M=(抑郁 OR 抑郁症 OR 抑郁状态 OR 抑郁情绪)) AND (M=(同型半胱氨酸 OR 同型半胱氨酸血症 OR Hcy)) AND (M=(认知 OR 认知功能 OR 认知损害OR 认知障碍)) |
