# Supplementary figures and images for: Homocysteine and cognitive function in depression: a systematic review and meta-analysis
Source: Front Psychiatry. 2026 May 13;17:1798998. doi: 10.3389/fpsyt.2026.1798998 (PMC13212284; doi:10.3389/fpsyt.2026.1798998)

**Supplementary File 2:** The overall evidence quality for outcome measure


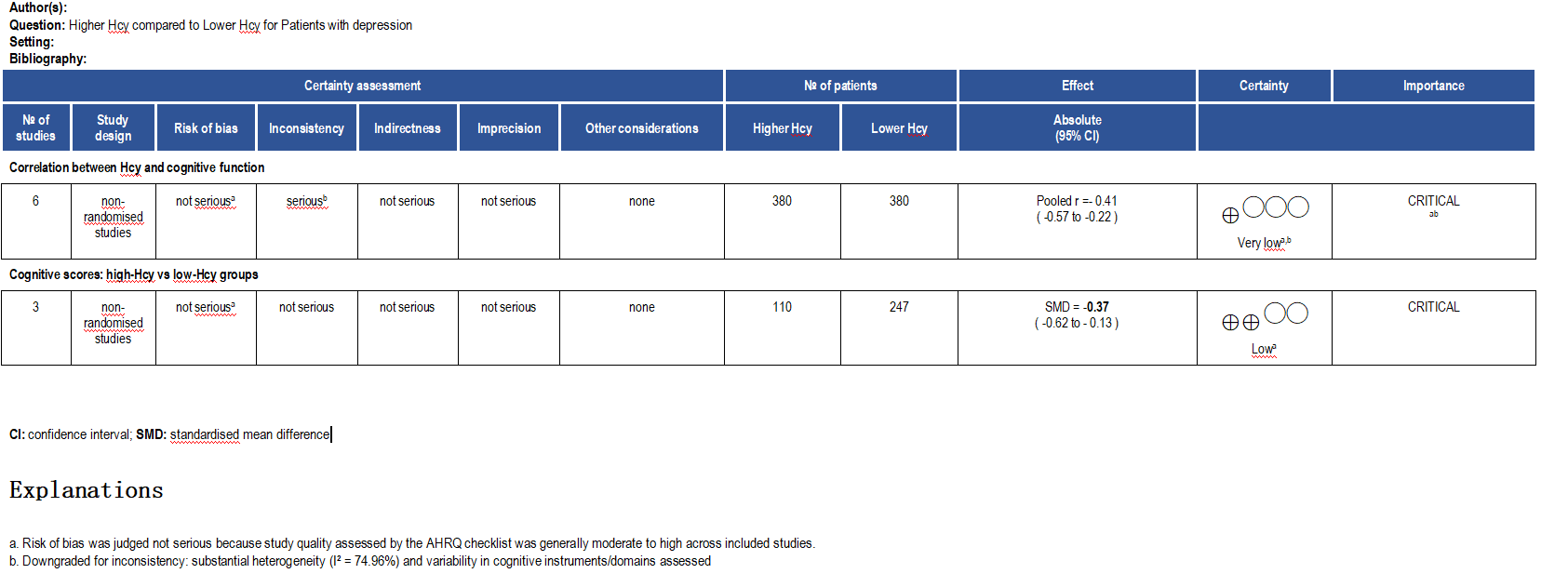


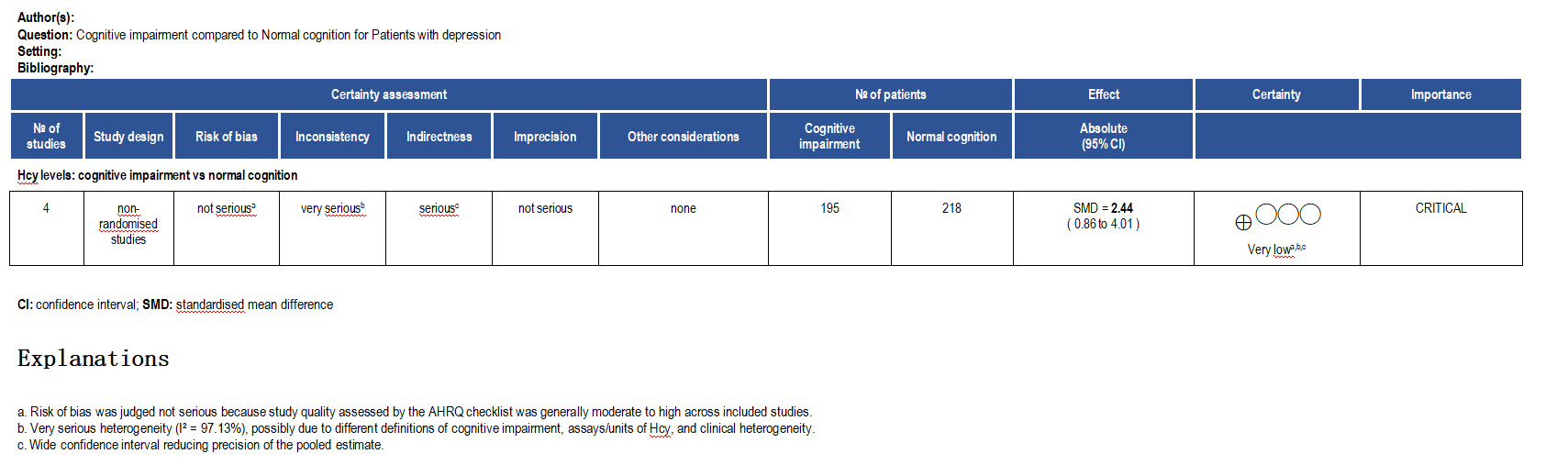

Supplement: Supplementary file 2 [file Table2.docx]
