# Supplementary material for: Homocysteine and cognitive function in depression: a systematic review and meta-analysis
Source: Front Psychiatry. 2026 May 13;17:1798998. doi: 10.3389/fpsyt.2026.1798998 (PMC13212284; doi:10.3389/fpsyt.2026.1798998)
Supplement: Supplementary file 3 [file Table3.docx]

**Supplementary File 3**: Leave-one-out sensitivity analysis for the correlation meta-analysis

| **omitted** | **pooled_r** | **lb_r** | **ub_r** | **I^2^** | **tau^2^** |
| --- | --- | --- | --- | --- | --- |
| Bell1992 | -0.401 | -0.579 | -0.186 | 80.109 | 0.058 |
| Fan2025 | -0.438 | -0.620 | -0.211 | 78.000 | 0.062 |
| Guo2021 | -0.303 | -0.408 | -0.190 | 0.000 | 0.000 |
| Xu2025 | -0.446 | -0.622 | -0.226 | 76.393 | 0.058 |
| Zhou2021 | -0.447 | -0.623 | -0.227 | 75.465 | 0.057 |
| Zhu2020 | -0.415 | -0.608 | -0.176 | 79.490 | 0.067 |
